# "Caught in Each Other’s Traps": Factors Perpetuating Incentive-Linked Prescribing Deals Between Physicians and the Pharmaceutical Industry

**DOI:** 10.34172/ijhpm.2024.8213

**Published:** 2024-04-27

**Authors:** Mishal Sameer Khan, Afifah Rahman-Shepherd, Muhammad Naveed Noor, Amna Rehana Siddiqui, Catherine Goodman, Virginia Wiseman, Afshan Khurshid Isani, Wafa Aftab, Sabeen Sharif, Sadia Shakoor, Sameen Siddiqi, Rumina Hasan

**Affiliations:** ^1^Faculty of Public Health and Policy, London School of Hygiene and Tropical Medicine, London, UK.; ^2^Department of Pathology and Laboratory Medicine, Aga Khan University, Karachi, Pakistan.; ^3^Department of Community Health Sciences, Aga Khan University, Karachi, Pakistan.; ^4^Centre for Social Research in Health, University of New South Wales, Sydney, NSW, Australia.; ^5^The Kirby Institute, University of New South Wales, Sydney, NSW, Australia.; ^6^TB Control Program, Health Department, Government of Sindh, Sindh, Pakistan.

**Keywords:** Private Healthcare, Healthcare Quality, Conflict of Interest, Pharmaceutical Marketing, Pakistan, Mixed Methods

## Abstract

**Background:** Despite known adverse impacts on patients and health systems, "incentive-linked prescribing," which describes the prescribing of medicines that result in personal benefits for the prescriber, remains a widespread and hidden impediment to quality of healthcare. We investigated factors perpetuating incentive-linked prescribing among primary care physicians in for-profit practices (referred to as private doctors – PDs), using Pakistan as a case study.

**Methods:** Our mixed-methods study synthesised insights from a survey of 419 systematically sampled PDs and 68 semi-structured interviews with PDs (n=28), pharmaceutical sales representatives (SRs) (n=12), and provincial and national policy actors (n=28). For the survey, we built a verified database of all registered PDs within Karachi, Pakistan’s most populous city, administered an electronic questionnaire in-person and descriptively analysed the data. Semi-structured interviews incorporated a vignette-based exercise and data was analysed using an interpretive approach.

**Results:** Our survey showed that 90% of PDs met pharmaceutical SRs weekly. Three interlinked factors perpetuating incentive-linked prescribing we identified were: gaps in understanding of conflicts of interest and loss of values among doctors; financial pressures on doctors operating in a (largely) privately financed health-system, exacerbated by competition with unqualified healthcare providers; and aggressive incentivisation by pharmaceutical companies, linked to low political will to regulate an over-saturated pharmaceutical market.

**Conclusion:** Regular interactions between pharmaceutical companies and PDs are normalised in our study setting. Progress on regulating these is hindered by the substantial role of incentive-linked prescribing in the financial success of physicians and pharmaceutical industry employees. A first step towards addressing the entrenchment of incentive-linked prescribing may be to reduce opposition to restrictions on incentivisation of physicians from stakeholders within the pharmaceutical industry, physicians themselves, and policy-makers concerned about curtailing growth of the pharmaceutical industry.

## Background

**Key Messages**

**Implications for policy makers**

- Incentive-linked prescribing, which is the prescribing of medicines that result in personal benefits for the prescriber, is widespread and detrimental to patients and the health system.
- In line with studies in other settings, we found that 90% of private doctors (PDs) meet pharmaceutical sales representatives (SRs) on a weekly basis, and that accepting or soliciting incentives (“deal-making”) is normalised.
- We identified three priorities to tackle incentive-linked prescribing: addressing professionalism and gaps in doctors’ understanding of conflicts of interests, understanding financial pressures on doctors and reducing unethical marketing practices by pharmaceutical companies.
- Challenges to addressing incentive-linked prescribing include insufficient evidence on successful policy responses and vested interested of key stakeholders—policy-makers, pharmaceutical companies, and doctors—who benefit from incentive-linked prescribing; this results in low political will to implement existing regulations prohibiting incentive-linked prescribing deals between doctors and pharmaceutical companies.

**Implications for the public**

Incentive-linked prescribing occurs when doctors accept personal benefits from pharmaceutical companies in return for prescribing medicines specified by the companies; these medicines are often unnecessary and overly costly for patients. Reducing incentive-linked prescribing is crucial so that patients can be sure they are receiving unbiased medical advice that is in their best interest. Incentive-linked prescribing financially benefits doctors and the pharmaceutical industry. Our interviewees highlighted that medical education does not effectively prepare doctors to navigate relationships with the pharmaceutical industry, especially when tempting offers are made by pharmaceutical sales representatives (SRs). Effectively formulating or implementing regulations to control incentive-linked prescribing may first require reducing opposition to change from doctors, the pharmaceutical industry and politicians.

In many Asian countries, the majority of patient consultations are with for-profit healthcare providers who seek to make a financial gain from their services.^1^ There is growing evidence that healthcare providers’ motivation to generate profits can result in the overuse of health services or products.^2-7^ Essentially, for-profit healthcare providers experience a conflict of interest because their professional judgment concerning a primary interest (the patient’s welfare) is at risk of being unduly influenced by a secondary interest (financial gain).^8,9^

 Financial relationships between pharmaceutical companies and healthcare providers have been controversial for decades, partly owing to concerns about the conflict of interest created.^10^ Evidence suggests that doctors and other healthcare providers responsible for prescribing medicines can be strongly influenced by pharmaceutical company marketing, which often involves giving gifts (or bribes) in return for meeting prescribing targets of their marketed medicines.^11^ Our study investigates incentive-linked prescribing, which occurs when providers receive monetary or non-monetary gains from pharmaceutical companies for prescribing specific medicines. Incentive-linked prescribing is a challenge in a range of countries, and contributes to the overuse of medications globally.^11-16^ It results in higher costs for patients, with a differential impact on the poorest, and exposes them to risks from adverse effects, ultimately reducing their trust in the healthcare system.^3,17-19^

 Evidence from qualitative studies in low- and middle-income countries (LMICs), including Pakistan, Bangladesh, India, and Brazil indicates that pharmaceutical company incentivisation of doctors for prescribing medications, in ways that would be considered unethical by regulators, is normalised practice.^17,19,20^ Factors that support inappropriate relationships between pharmaceutical companies and doctors include weak implementation of regulations as well as doctor and pharmaceutical industry support for continuing incentivisation.^20,21^ Being a powerful stakeholder group, doctors’ perceptions and attitudes towards relationships with the pharmaceutical industry and incentive-linked prescribing can influence the success of policies to reduce the impact of the pharmaceutical industry on prescribing practices^22^; doctors’ awareness, attitudes, and reasons for engaging in incentive-linked prescribing are therefore important to understand.^23^ However, because for-profit healthcare providers are often poorly monitored and documented in LMICs, large quantitative and mixed methods studies of their attitudes and practices are rare.^24^

 We investigate incentive-linked prescribing by doctors and conflict of interest related to pharmaceutical marketing Pakistan (Box 1). This is an insightful setting due to the unusually large number of registered medicinal products and dominance of Pakistani companies producing generic drugs that are marketed as “branded generics.”^25^ A branded generic market—especially one that is overcrowded with companies and products—creates marketing pressure, which encourages pharmaceutical companies to incentivise doctors as a means to compete for the relatively small patient market compared to the number of medicines being sold.^25,26^ Despite the availability of international and national codes of ethical conduct for the pharmaceutical industry and doctors (Box 1), enforceability remains a challenge, particularly in countries where regulatory bodies are under-resourced like Pakistan.^14,27,28^ We focus on for-profit primary care doctors (referred to as private doctors [PDs] going forward) as they are the first point-of-contact for the majority of the population in Pakistan, and they typically operate small clinics as entrepreneurs, without being bound by prescribing guidelines that often apply to doctors operating secondary or tertiary care hospitals.^19,29-31^ Our two study objectives were to assess knowledge and attitudes on conflicts of interest and the acceptance of incentives for prescribing among a systematic sample of PDs in Karachi and to elucidate factors perpetuating incentive-linked prescribing from the perspective of doctors, pharmaceutical sales representatives (SRs), and a range of health policy actors.

**Box 1. Overview of the Study Setting**

Pakistan is the fifth most populous country in the world and the proportion of healthcare sought in the for-profit private sector is one of the highest globally (estimated at over 70%).^24^ Funding for public services has been declining since the early 1990s, contributing to a dominant private healthcare sector consisting of qualified and unqualified providers.^24^ Out-of-pocket expenditure accounts for over 50% of current health expenditure in Pakistan.^32^ It has a burgeoning pharmaceutical sector and, like many countries, there is a problem of excessive and inadequate access to medicines.^33,34^ More than 700 pharmaceutical companies operate in Pakistan, of which less than 30 are multinational companies, and there is a history of challenges in regulating interactions between the pharmaceutical industry and healthcare providers.^19,35^ Pakistan has unusually large number of registered medicinal products; the upper range is estimated to be as high as 88 000, compared to 20 000 in the United States. This high number of brands being marketed per active ingredient results in strong competition between companies to have their medicines prescribed by doctors.^22,28^ International guidelines to regulate pharmaceutical promotion and marketing by the WHO and the International Federation of Pharmaceutical Manufacturers and Associations are reportedly adopted by some companies in Pakistan.^19^ Nationally, the Drug Regulatory Authority of Pakistan revised their rules on ethical pharmaceutical marketing in the health sector in 2021, and the Pakistan Medical and Dental Council have a *Code of Ethics of Practice for Medical and Dental Practitioners* published in 2011. According to all these policies, pharmaceutical companies are explicitly prohibited from giving incentives such as cash, gift cards, food, gift baskets, flowers or any type of branded promotional goods to healthcare professionals, who are generally defined as any member of the medical, dental, pharmacy, or nursing professions, or any health personnel involved in recommending, prescribing, purchasing, supplying, dispensing or administering a pharmaceutical product. Healthcare professionals are likewise prohibited from accepting such incentives in exchange for prescribing. In spite of this, several studies have evidenced how common incentive-linked prescribing is in Pakistan.^19,21,27^ The extent to which these practices occur has been described as “an acceptable norm,” one that is challenging to undo.^19,35^

-----------------

Abbreviation: WHO, World Health Organization.

## Methods

 Our mixed methods study synthesised data from a cross-sectional survey and semi-structured interviews conducted between October 2021 and June 2022.

###  Quantitative Data Collection and Analysis

 To conduct the cross-sectional survey, we first had to build a sampling frame of primary care physicians engaged in for-profit practices ie, in private (non-government run) clinics managed as businesses generating an income from fees paid in exchange for medical services, with and without additional work in the public sector. We obtained a list of 1695 healthcare providers in Karachi from the provincial regulatory body and applied our eligibility criteria to filter for those that had received formal training in medicine (Bachelor of Medicine, Bachelor of Surgery, MBBS), were registered with the Pakistan Medical Commission, and were working as PDs as per our definition. This resulted in 1185 potentially eligible study participants who were contacted by phone to validate their information. A total of 763 physicians were excluded after validation: nine were not contactable, 18 refused to verify their information over the phone, and 736 did not meet our definition of a PD based on information provided during the phone verification (ie, provided specialist care rather than primary care, operated a non-profit clinic, worked within a tertiary care facility, or were no longer registered with the regulators). We derived 422 PDs from our sampling frame, of which 419 (99.2%) consented to participate in the survey.

 Our electronic questionnaire was completed by PDs in their clinics in Urdu or English following an in-person explanation from data collectors trained by members of the team. Questions covered demographics, (medical) educational background, interactions with pharmaceutical SRs, knowledge of conflict of interest, and attitudes towards different incentives. We also explored PDs’ values and religiosity by adapting a Moral Sensitivity questionnaire developed in Turkey.^36^ To assess knowledge, we reviewed key international and national guidelines on the interactions between physicians and the pharmaceutical industry, and formulated “True or False” statements based on their content. For example, World Health Organization (WHO) guidelines state that SRs should not offer inducements to prescribers, and prescribers should not solicit such inducements. In relation to events and meetings, International Federation of Pharmaceutical Manufacturers and Associations guidelines prohibit any entertainment or other leisure or social activities to be provided or paid for by pharmaceutical companies.^37,38^ To assess attitudes, we listed different types of incentives and asked whether these incentives were “acceptable” or “unacceptable” to take in return for meeting targets for prescribing specific medications. When scoring the answers, we considered all types of incentives to be unacceptable when linked to prescribing targets.

 Data were initially exported to MS Excel for translation to English (where needed) and cross-checked by bilingual research team members. Data cleaning and descriptive analyses were then conducted in STATA (version 17).

###  Qualitative Data Collection and Analysis

 We conducted 68 interviews with PDs in Karachi (n = 28), pharmaceutical SRs from multinational, national and franchise companies (n = 12), and health policy actors working at the national and provincial levels (n = 28). Health policy actors were defined as individuals with direct or indirect influence on health policy relating to pharmaceutical company and healthcare provider interactions. This included manager level staff of regulatory bodies and the pharmaceutical industry, as well as media and communication experts, ethicists, and officials of professional medical associations. Snowball sampling was used to identify policy actors: an initial group of ten were contacted through the research team’s networks, then 18 individuals recommended by the first group of interviewees were approached for further interviews. All interviews were conducted in Urdu and in-person, where possible. We continued approaching interviewees in each category until no new themes appeared in interviews, which indicated data saturation.^39^ Interviews typically lasted 60 minutes and were audio-recorded for transcription, bar one upon the interviewee’s request. Transcripts were translated from Urdu to English and quality checked by bilingual members of the research team. Each interview involved a piloted vignette-based discussion introduced at the beginning of the interview to explore unnecessary prescribing of medicines (Box 2), and used a topic guide with semi-structured questions on pharma-physician dynamics and the feasibility of different interventions to improve prescribing practices designed by the authors.

**Box 2. Vignettes Presented to Initiate Discussion in the Semi-structured Interviews**

A male physician aged 35, with an MBBS degree, runs a solo practice clinic in Karachi and is the only financial provider for his wife, three young children and elderly parents. He finds that the income he earns from patient consultations alone is not enough to provide for his family. When a female patient aged 50 comes to see him for symptomatic relief of a runny nose, sneezing, and itchy eyes (without fever) for the past two days, he prescribes antibiotics even though the patient does not ask for any specific medications. He knows that antibiotics are not necessary in this case. He undertakes irrational prescribing of antibiotics approximately ten times per week.

Now I will describe why he prescribes antibiotics unnecessarily and please tell me your views (interviewee shows cards with information below written on them one by one).

1. Doctor Ahmed – prescribes the antibiotics because he receives gifts from a pharmaceutical company when he meets their targets at the end of each month
2. Doctor Imran – prescribes the antibiotics so that a pharmaceutical company will pay for him to attend a conference that he cannot afford to pay for himself.
3. Doctor Ijaz – tells patients to buy the prescribed antibiotics from his friends’ shop because his friend gives him a percentage of the sales of every medicine.
4. Doctor Asif – prescribes antibiotics because he is afraid that if he gives the patient no medicine the patient will be unsatisfied and tell others in the community not to visit his clinics.

-----------------

Abbreviation: MBBS, Bachelor of Medicine, Bachelor of Surgery.

Data were analysed thematically using an interpretive approach such that themes are supported by excerpts from the raw data.^40^ Two authors (SabS and ARS) independently coded the same ten transcripts in NVivo (version 12) line-by-line independently to develop a preliminary codebook (coding tree), which was tested and refined by two other authors (MSK and WA). After authors reached a consensus on the final codebook, SabS and ARS coded each of the remaining transcripts deductively in NVivo. The final codebook acted as a guide to identify themes presented in the analysis. The abbreviations “PD,” “SR,” and “PA” are used to indicate excerpts from private doctors, pharmaceutical SRs, and policy actors, respectively.

## Results

###  Participant Demographics and Overview of Findings

 All 419 PDs enrolled in the study were surveyed. Characteristics of study participants are presented in Table and available in recent publications.^21,41^ We found that 90% of PDs met with SRs at least once a week, with 15% of PDs meeting more than 25 SRs per week.

**Table:** Characteristics of Private Doctors From the Survey

**Characteristics**	**Study Sample** **(n = 419)**
Age (y), mean (SD)	54.5 (10.1)
Gender, male (%)	361 (86.2)
Additional professional qualification^a^ (%)	74 (17.7)
Years of experience, mean (SD)	29.8 (9.6)
Self-reported number of patients seen daily (%)	
<25	115 (27.4)
25-50	188 (44.9)
51-75	61 (14.6)
>75	55 (13.1)
Self-reported number of meetings with pharmaceutical SRs weekly (%)	
0	42 (10.0)
<25	315 (75.2)
25-50	43 (10.3)
>50	19 (4.5)

Abbreviations: SRs, sales representatives; SD, standard deviation.

 Our analysis identified a range of factors contributing to, and sustaining, incentive-linked prescribing. In the following section, we integrate key findings from the survey and semi-structured interviews and present these under the following broad themes: education factors, financial factors, and market and policy factors.

###  Gaps in Understanding of Conflicts and Loss of Professional Values

**Figure 1:**
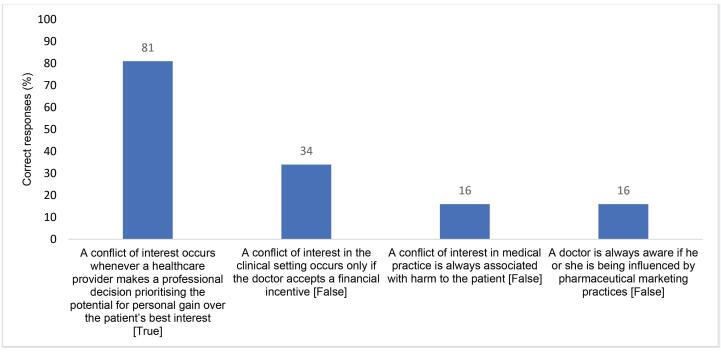
Private Doctors Survey Responses on Knowledge of Conflicts of Interest Between Doctors and Pharmaceutical Companies (correct response indicated in square brackets) (n = 419).

 Based on the survey, although the majority of PDs (81%) knew that a conflict of interest occurs whenever there is a risk that a provider prioritises their personal gain over the patient’s best interest, two-thirds (66%) do not associate taking non-financial incentives—which were commonly mentioned during interviews—with conflict of interest (Figure 1). A very high proportion also did not know that conflicts of interest can be present without causing any physical harm to the patient (84%), for example through the prescription of (unnecessary but often costly) vitamins in exchange for benefits from pharmaceutical companies, or that doctors might be unaware that they are being influenced by pharmaceutical marketing (84%) (Figure 1).

 The knowledge gaps identified in the survey were consistent with interviewees’ recognition of the inadequacies of current medical education and professional development on conflict of interest. Numerous policy actors discussed the insufficient attention to pharma-physician relationships in medical education and training; five also highlighted that pharmaceutical companies may fund medical colleges and continuing medical education workshops, implying that this may compromise the impartiality of education on topics such as pharma-physician relationships. Half of the SRs also confirmed that pharmaceutical companies provide continuing medical education for doctors.

*“So, when we are going to educate [doctors], we also need to check every institute in terms of how much funding they are getting from pharmaceutical companies and what items they have had installed in their rooms or their offices from the pharmaceutical companies”* (PA).

**Figure 2:**
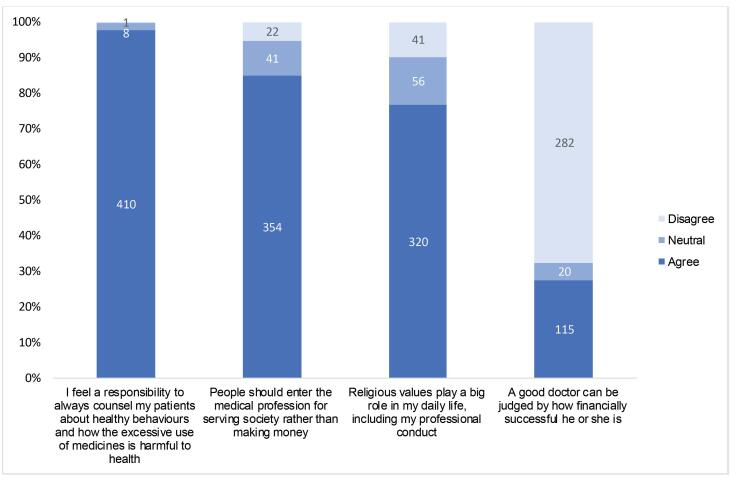
Private Doctors Survey Responses to Questions on Moral Sensitivity (n = 419).

 Although the majority of PDs (85%) agreed that they should enter the medical profession to serve society, rather than to make money (Figure 2), many PDs and policy actors expressed concerns about the loss of key professional values associated with practicing medicine, such as honesty and integrity. They described a profit-making mindset in which doctors are increasingly preoccupied with maximising their earnings, rather than service and justice, and attributed this decline to the commercialisation of medical education and healthcare provision. Three policy actors and one SR asserted that improvements in medical education and training could not change the deficits in morality or a lack of personal responsibility.

*“You see, it is all commercial now. Forget the concept that ‘he will become a doctor and is going to serve, etc.’ That mentality is finished. Today, it is all commercial”* (PD).

*“[…] on ethics, however much you teach, the nature of the person will not change, as when a person becomes greedy then he does not remember what he has studied. If he wants money, he just wants money [...]” *(PA).

###  Financial Circumstances of Physicians and Competition Among Providers 

**Figure 3:**
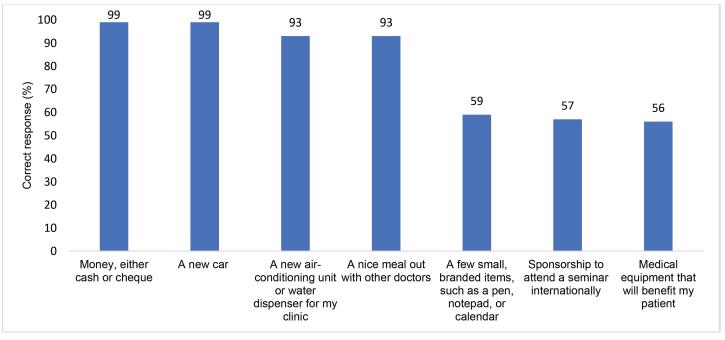
Private Doctors Survey Responses on Attitudes Toward Different Types of Incentives in Return for Prescribing Pharmaceutical Companies’ Products (correct if participant responded “unacceptable”) (n = 419).

 While over 90% of surveyed PDs agreed that most incentives, especially in the form of cheque, cash, and commission, were unacceptable to take from pharmaceutical companies (Figure 3), almost all interviewed PDs and SRs reported how common and entrenched the exchange of financial (and non-financial) incentives are.

 We found contrasting views on whether physicians were passive “victims” of incentivisation owing to their financial needs combined with aggressive marketing by pharmaceutical companies, or whether physicians play an active role in driving a culture of incentivisation. The PDs we interviewed tended to report that the increased focus on profit-making was largely influenced by the pharmaceutical industry’s marketing, while SRs and policy actors tended to report that doctors were encouraging these practices because it benefits them. These contrasting perspectives are illustrated in the quotes below.

*“Why would doctors refuse [pharmaceutical companies] when they are getting money by just being there…”* (PD).

*“If the doctor needs cash, then 20% is given in the form of cash. If their need is a tour, then it can be in the form of tour. If the need is for planning an anniversary, then it will be in that form. So the doctor is tells you what they need” *(SR).

 Nine PDs also spoke about the need to generate sufficient income, which they thought led their colleagues to adopt profit-maximizing behaviours, even though all surveyed PDs agreed that doctors have a responsibility to give patients the best care even if this reduces their income (Figure 2). Other PDs disagreed that a doctors income was insufficient to such an extent that financial support from the pharmaceutical industry was a necessity. Similarly, several SR and policy actors did not think there was an income challenge for doctors and that this profit-maximizing behaviour was instead a form of “*greed*.”

 PDs described three main sources of financial stress. First, (private) medical education being incredibly costly; they reflected that young doctors starting their careers seek to recoup investments in medical education quickly. Second, that established doctors are expected to have a high social and financial status, which can force doctors to live beyond their means. Third, perceptions that patients have more confidence in doctors who are outwardly financially successful. Though most surveyed PDs (68%) did not believe that a good doctor can be judged by their financial successes, almost one third equated professional success with financial success (Figure 2).

 Finally, a few PDs commented on the proliferation of providers, especially unqualified providers, and the competition this creates for patients. One suggested that SRs do not discern between qualified and unqualified providers, meaning that unqualified providers are also being incentivized to meet prescribing targets, further exacerbating the level of competition. Several interviewees suggested that unqualified providers draw patients away from qualified providers, which, in turn, compels qualified providers to generate additional income through unnecessary prescriptions.

*“When there is competition in any neighbourhood, where a doctor and four quacks are sitting, then the patient sees from where he will get better quickly. There, the doctor out of compulsion gives more antibiotics [...]”* (PD).

###  Aggressive Incentivisation by Pharmaceutical Companies and Lack of Political Will to Curtail Industry Growth

 Six SRs suggested that many colleagues, particularly those working for national companies, face pressure from management “to meet targets by all means” and can experience financial insecurity because their salary is often directly linked with monthly sales. Interviewees said that SRs use aggressive marketing tactics to engage more PDs in target-linked prescribing, such as visiting doctors very frequently, building a social relationship with them, and offering attractive incentives that appeal to the financial and non-financial needs of PDs. While the majority of surveyed PDs found incentives such as money, a new car, a new air-conditioning unit, or a nice meal unacceptable, 40%-50% of them considered other types of incentives acceptable such as small, branded items, sponsorship for educational events, and medical equipment (Figure 3). Interviewees also highlighted that those pharmaceutical companies that spend more on incentive packages have a competitive advantage over other companies. In such a saturated market, interviewees described incentivisation as integral to pharmaceutical companies’ business model. One SR described it as companies getting “*caught in each other’s trap*,” implying that there is an upward cycle to incentivisation practices.

*“[The] offer from the person selling the medicine or a demand from the person prescribing the medicine, in both situations [it is] to earn more money. This has become a business strategy in our profession since the early ‘80s [...]” *(PA).

 Interviewees suggested that a disproportionate amount of power is held by the pharmaceutical industry, with substantial lobbying strength to influence policy-makers. As one policy actor highlighted in the quote below, an important reason for the industry’s power is that it is seen by politicians as an important positive contributor to the country’s economy, which it uses as political leverage. Additionally, interviewees highlighted the lack of powerful lobby groups to counter the culture of incentivisation, and the general reluctance of policy-makers to oppose powerful stakeholder groups during their terms in office, such as the pharmaceutical industry and professional medical associations.

*“[…] doctors are making money off the patients, but at the end of the day, it adds to the ‘vitality’ of the pharmaceutical industry. […] So, the government doesn’t see anything [in terms of marketing violations], they only hear what one lobby is saying to them […] [That] the pharmaceutical industry does not like to be restrained too much in marketing” *(PA).

 Although a minority, it was striking that three PDs and two policy actors referred to the industry as a “*mafia.*” Several interviewees across all groups alluded to a wider culture of corruption, which they thought helped create an enabling environment to normalise incentivisation.

*“I think we need to have a very stringent code of ethics. [In] Pakistan, very few companies that comply with it because, if you go and listen, those [regulators] are the ones who are asking for a bribe. So, the implementers are the biggest demanders as well” *(PA).

 These factors were believed to result in a lack of political will to address the problem of incentive-linked prescribing. Interviewees spoke of insufficient resources allocated to the agencies responsible for regulating the pharmaceutical industry and their marketing practices, and the lack of mechanisms to effectively monitor rule-breaking of both pharmaceutical companies and healthcare providers, such as auditing of prescriptions, watchdogs, and whistleblowing processes. Interviewees also questioned which government agency, for example the pharmaceutical regulator, healthcare quality commissions or physician licencing bodies, would be responsible for implementing such mechanisms, given the gaps in ownership of this issue and in regulatory capacity. Regulatory agencies were described as ‘lacking teeth’ and seen as relatively weak in terms of power compared to the pharmaceutical industry and doctors.

## Discussion

**Figure 4:**
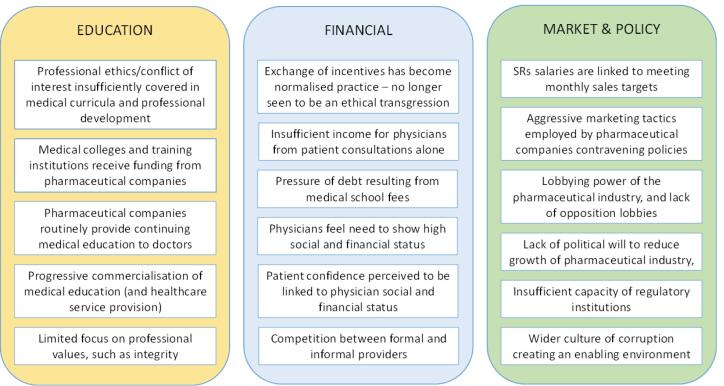
Education, Financial, and Market and Policy Factors Influencing Physicians’ Decision-Making. Abbreviation: SRs, sales representatives.

 Incentive-linked prescribing is a key form of over-provision of healthcare, which is known be widespread, harmful and increasing around the world.^7,42^ Our research identified three interlinked factors that perpetuate incentive-linked prescribing: gaps in effective medical education on conflicts of interest; (perceived) financial pressures on PDs, necessitating the acceptance of monetary or non-monetary incentives from the pharmaceutical industry; and low political will to enforce marketing regulations or over-saturation of the pharmaceutical market (Figure 4).

 The entrenchment of incentive-linked prescribing in society has implications for patients, as there is strong evidence that pharmaceutical company payments to healthcare professionals are associated with increases in prescribing of the incentivising company’s drug and costs borne by patients.^43,44^ In keeping with our findings, the relatively limited evidence from LMICs indicates that interactions between physicians and SRs are very common, with SRs visiting up to 90% of physicians in other countries as well.^11^ Further, studies from countries as diverse as Uganda and Peru also report that relationships with pharmaceutical companies strongly influence physicians’ attitudes and practices.^45,46^ In light of the pervasiveness of incentive-linked prescribing worldwide, it is important to reflect on the barriers to making sustained progress towards reducing this practice.

 A critical challenge we illuminated is that the key stakeholders involved—policy-makers, domestic and international pharmaceutical companies, and PDs—often have vested interests in this practice continuing. This may manifest in the issue being insufficiently prioritised at the agenda setting stage, or policy formulation and implementation of regulations being weak.^21^ Strong stakeholder interests in maintaining the status quo may explain why the relationship between pharmaceutical companies and PDs has been a contentious and insufficiently managed policy issue for over 60 years globally,^10^ despite studies highlighting the need for policy and education interventions.^23,47^

 The lack of political will to curtail the pharmaceutical industry’s growth has been found in other settings as well.^22,48,49^ Our qualitative data indicates that the lack of political will partly stems from politicians’ motivations to support the growth of the domestic pharmaceutical industry and is influenced by industry lobbying against limitations on marketing or proliferation of active companies. These findings are consistent with a recent analysis in Pakistan highlighting the “entrenchment of industry interests within governmental institutions.”^50^

 Because prescribing deals between doctors and the pharmaceutical industry have gradually become normal practice in Pakistan, with limited discourse to challenge this norm, another reason for policy-makers’ inertia on incentive-linked prescribing may be concern about opposition from doctors when doctors’ interactions with the pharmaceutical industry are constrained. Here, it may be useful to consider governance of the private sector as a continuum evolving from soft approaches (persuasion, dialogue, and voluntary activities) to harder regulations with associated penalties for non-compliance.^51^ Exploration of education and persuasion-based approaches may be especially relevant when doctors lack awareness of the impact of subtle forms of conflicts of interest; for example, in our study population, there was insufficient understanding of the implications of incentive-linked prescribing of products that are perceived to have no side effects for patients (eg, vitamins). However, our study and a systematic review analysing knowledge and attitudes of doctors regarding interactions with the pharmaceutical industry, identified a challenge to education-based approaches – doctors’ beliefs that they are personally immune from influence on prescribing practices, while recognising that conflict of interest may be problematic for colleagues.^23^ Another approach to reduce opposition to stronger regulatory controls on relationships between doctors and pharmaceutical companies is to convene prominent allies that would support change; for example, this has been done for alcohol related policy change.^52^ Multinational and domestic pharmaceutical companies that follow ethical marketing practices may be a group of stakeholders willing to support a shift away from incentivisation, since it is harder for these companies to continue to abide by ethical codes of conduct when the majority of their competitors have abandoned them.

 When looking towards implementation of harder regulations to address incentive-linked prescribing, it is important to consider that harder approaches, including sanctions such as fines for pharmaceutical companies and licence cancellation of doctors, have not demonstrated consistent changes in prescribing practice in a range of high-income countries.^53,54^ Increased transparency through mandatory disclosures about financial ties between health professionals has had mixed results and there is only weak evidence in favour of regulations limiting interactions (meetings, promotional materials, free samples) between pharmaceutical representatives and doctors.^55,56^

###  Strengths, Limitations, and Future Research

 As mentioned earlier, an important contribution of our mixed-methods study is the quantitative data collected using a verified, systematic sampling frame of primary care doctors engaged in for-profit practices. The focus on primary care doctors, explained earlier, raises questions about generalisability of our findings to specialist doctors. Evidence from Pakistan indicates that specialist physicians are also commonly engaged in incentive-linked prescribing deals, although we were not able to compare pharmaceutical company engagement with primary versus specialist care physicians^57,58^ Comparing knowledge, attitudes, and perceptions of incentive-linked prescribing across different types of medical providers in Pakistan and elsewhere may insightful. For example, it may be that doctors working only in the public sector or in non-profit clinics have a less financially motivated mindset than the population we studied and those working in hospitals may be constrained by guidelines on prescribing or meeting with pharmaceutical SRs. Our survey questionnaire had to be brief in order for PDs to complete it and our quantitative results may have missed some domains of knowledge and attitudes toward incentive-linked prescribing. The moral sensitivity questions that we adapted for use in Pakistan have not been validated in this context and as such, should be interpreted with some caution. Future studies expanding the survey questions on knowledge and attitudes, and to validate the moral sensitivity questionnaire in this context to better understand the association of “morality” and “religiosity” (to the extent that these can be measured) with ethical medical practice may be insightful. A strength of our qualitative data is the mixture of stakeholders represented, including difficult-to-access SRs. We were also able to analyse interviews in Urdu and English such that nuance in Urdu phrases were not lost. Owing to the sensitive nature of the topics being discussed and our team potentially being perceived as “outsiders” to the medical profession, we recognise that interviewees—particularly PDs and SRs—may have been guarded in their responses, underplaying the extent to which they are aware or engage in incentive-linked prescribing, or exaggerating the role of another stakeholder in order to shift blame.

## Conclusion

 As the role of private health financing and for-profit healthcare providers increases around the world, it is critical to address the well-documented over-prescribing of medicines because of incentivisation by pharmaceutical companies. Our study highlights several impediments to regulatory approaches and international codes of conduct that restrict the nature or frequency of interactions between physicians and SRs: the pharmaceutical industry is well-resourced relative to regulators; doctors are typically benefiting financially from incentivisation with limited reflection on the downsides of this practice; and policy-makers typically do not want to curtail economic growth supported by the pharmaceutical industry. Although these interlinked barriers are complex to address, it is crucial to reduce stakeholder opposition to regulatory controls on pharmaceutical company engagement with healthcare providers, and to increase political will to reduce incentive-linked prescribing.

## Acknowledgements

 We would like to acknowledge the study participants for their generous contributions in time and insight toward understanding this complex and challenging issue. We would also like to acknowledge Nina van der Mark for assisting in the final stages of preparing the manuscript.

## Ethical issues

 We received ethics approval from the Aga Khan University Ethics Review Committee (reference 2020-4759-1129), the National Bioethics Committee of Pakistan (reference 4-87/NBC-582/21/1364), and the London School of Hygiene and Tropical Medicine Research Ethics Committee. Formal written consent was obtained from all study participants.

## Competing interests

 Authors declare that they have no competing interests.

## Disclaimer

 The datasets generated and analysed during the current study are not publicly available owing to the potentially sensitive nature of our research on conflict of interest, and the legal and/or professional ramifications for physicians and pharmaceutical companies for contravening codes of ethical practice. Some additional data may be available from the corresponding author on reasonable request.

## Reflexivity statement

 The following authors worked for the Aga Khan University, Karachi, Pakistan while this study was being conducted: Muhammad Naveed Noor (M), Assistant Professor, Amna Rehana Siddiqui (F), Professor, Wafa Aftab, Senior Instructor, Sabeen Sharif, Research Coordinator, Sadia Shakoor (F), Associate Professor, Sameen Siddqi (M), Professor, Rumina Hasan (F), Professor.

 The following authors worked for the London School: Mishal Sameer Khan (F), Professor and Catherine Goodman (F), Professor. The remaining authors were Virginia Wiseman (F), Professor and Afshan Khurshid Isani (F), employed by the TB Control Program, Health Department, Government of Sindh, Pakistan.

 This study is part of collaborative research on conflicts of interest and quality of healthcare in Pakistan ongoing since 2018; the specific objectives were co-designed with Pakistani researchers with input from policymakers and healthcare professionals, who highlighted incentivisation and ethical medical practice as a poorly addressed health systems challenge.

 All researchers involved in designing and collecting data were also involved in the analysis, write-up and authorship of this paper. Researchers were not known to any of the doctors or pharmaceutical SRs interviewed or surveyed but did have prior professional contact with several of the policy actors interviewed. We have gathered feedback from selected policy actors and are in the process of engaging doctors. Throughout the project we followed a 360-degree capacity building approach which recognises that researchers based in higher-income country institutions can learn from colleagues based in lower-income country institutions and vice versa.

## Funding

 This work was supported by the UK Research and Innovation (UKRI) under grant number MR/T02349X/1. The authors declare the funder played no role in the design, conduct of the study and preparation of this manuscript.
